# Deep learning in image segmentation for cancer

**DOI:** 10.1002/jmrs.839

**Published:** 2024-11-06

**Authors:** Robba Rai

**Affiliations:** ^1^ South Western Sydney Clinical School University of New South Wales Liverpool New South Wales Australia; ^2^ Liverpool and Macarthur Cancer Therapy Centre Liverpool Hospital Liverpool New South Wales Australia; ^3^ Ingham Institute for Applied Medical Research Liverpool New South Wales Australia

**Keywords:** Convolutional neural network, deep learning, semantic segmentation, U‐Net

## Abstract

This article discusses the role of deep learning (DL) in cancer imaging, focusing on its applications for automatic image segmentation. It highlights two studies that demonstrate how U‐Net‐ and convolutional neural networks–based architectures have improved the speed and accuracy of body composition analysis in CT scans and rectal tumour segmentation in MRI images. While the results are promising, the article stresses the need for further research to address issues like image quality variability across different imaging systems.

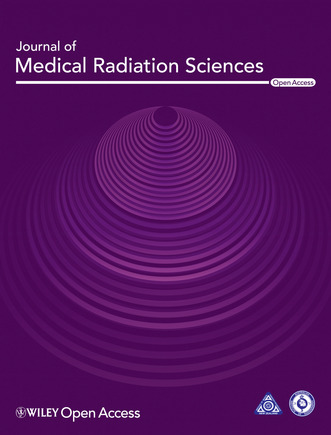

## Introduction

Cancer is one of the leading causes of death worldwide. In 2020, it accounted for nearly 10 million deaths globally.^1^ Medical imaging plays an important role in all stages of cancer management and treatment and is used to highlight the differences between normal tissue and regions suggestive of a neoplastic process.^2^ It is also used in a quantitative manner which is useful in characterising tumour types and measuring response during cancer treatment. The Quantitative Imaging Biomarkers Alliance (QIBA), a consortium of the Radiological Society of North America (RSNA), defines quantitative imaging as ‘the extraction of quantifiable features from medical images for the assessment of normal or the severity, degree of change, or status of a disease, injury or chronic condition relative to normal’.^3^ Advances in imaging technologies allow for early detection of diseases with precision, better tumour characterisation and assessment of changes in disease over time or in response to therapy.^4^

Imaging modalities such as computed tomography (CT), positron emission tomography (PET), single‐proton emission computed tomography (SPECT) and magnetic resonance imaging (MRI) are often used in cancer care for prediction, screening, biopsy guidance, staging, prognosis, therapy planning, treatment guidance, response assessment as well as detection of recurrence and palliation.^5^ Tumours are inherently spatially and temporally heterogeneous structures and can cause small populations of cancer cells that are resistant to treatment,^6^ resulting in treatment failure and drug resistance,^7^ tumour recurrence and poor prognosis^8^. Tumour heterogeneity in medical imaging can often be subjectively and qualitatively assessed by correlating varying grey levels and tumour borders with tumour invasiveness into surrounding stroma,^9^ oedema and regions of necrosis.^10^ Medical imaging is an essential diagnostic tool of all cancer treatment decisions and in certain circumstances can be used alone, without histopathology for oncological treatment decision‐making.^11^

The use of standard‐of‐care scans to acquire more quantitative information has become popular in recent years. The advantage of using standard‐of‐care scans is that they are in high abundance in hospital imaging archival systems, which is useful for retrospective research. However, there are challenges to using standard‐of‐care medical images for more quantitative purposes, due to variability in manual segmentation, image processing time and computing power and different imaging interpretations among physicians. Also, using images acquired from different imaging modalities across different centres and hospitals can inherently cause image quality differences which can impact image interpretation. Manual segmentation is a time‐consuming and laborious task and is prone to interobserver variability. In contexts such as radiation therapy where different imaging modalities are used to segment tumour volumes only and avoid healthy tissues, inaccurate segmentation can result in geographic miss during treatment and can potentially reduce local control, impacting overall survival outcomes.^12^ Standard‐of‐care scans can also be used to extract different types of imaging features such as texture and shape features; as in the field of radiomics, which can be used to characterise and classify tumours by phenotype.^6^ However, this requires high computational power and processing times to compute such a large volume of data.

Deep learning (DL) is emerging as a valuable tool for quantitative image analysis as it is a fast and efficient way to process copious amounts of data and allows for automation of these tasks.^13^ It has many applications for medical imaging, including but not limited to image segmentation, and has been shown to increase the accuracy and speed of image segmentation tasks for anatomical object localisation.^14^

This editorial aims to highlight two articles recently published in the Journal of Medical Radiation Sciences using DL to enhance different areas of cancer management, specifically segmentation of images on medical imaging scans.

## Deep Learning

Deep learning (DL) is one of the technologies in the field of artificial intelligence (AI) that has become a promising method to analyse large volumes of medical images in a more efficient and rigorous way. DL models are composed of multiple layers of artificial neurons. These layers progressively extract higher‐level features from raw input data, allowing the system to make predictions or decisions with greater predictive power. In DL, the layers include an input, one or more hidden layers and an output layer.^15^ The input layer receives the input data (training datasets) allowing the hidden layers to process the input data from weighted connections. During training, input data are provided to the model along with the correct output labels. The weights in the neural network are initialised (often randomly), and the network makes a prediction based on the input data. Initial training usually involves a large dataset that has been manually labelled by humans. For example, in supervised learning, humans may label images, sentences or data points with the correct category, which the model uses to learn.

These connections are referred to as deep neural networks (DNNs) and mimic the way the neural networks function in the human brain. The DNN systems continue to learn from input data due to their interconnected nature and this allows for more accurate results or predictions in the output layer.^16^

Applications of DL in medical imaging are vast and can significantly impact image analysis and interpretation. Some applications of DL in medical imaging include image classification, segmentation, detection, reconstruction and registration.

There are different types of DL architectures, including convolutional neural networks (CNNs), recurrent neural networks (RNNs), generative adversarial networks (GANs) and transformers.

## CNNs and U‐Nets

The most common type of architecture employed for medical imaging segmentation is CNNs.^16^ CNNs are designed for grid‐like data such as medical images. CNNs work by sliding a filter to detect features and identify patterns within images, creating spatial hierarchies in the data. This in turn will create a feature map that will serve as input for the next layer. This process will gradually create a hierarchical representation of the image. Each layer will have a specific filter that slides (or convolves) over the input image, and this creates a more complex feature map with more complex patterns. Pooling layers are used to down sample the data extracted from the convolution process, and this is performed to reduce the chance of overfitting. Overfitting occurs when a DL model performs very well on the training data but performs poorly on new, unseen data. This is problematic as the goal of DL is to build models that work well on new, unseen data – not just the data they have been trained on. In the final layers of the CNN, this model will make a final decision to classify that object (based on output from previous layers).

U‐Nets are a type of CNN that focuses on up sampling each operation to improve the resolution of the output and is commonly used for multiclass semantic segmentation. Instead of pooling layers that only down sample data in a traditional CNN as mentioned earlier, the U‐Net architecture will down sample and up sample data using a combination of convolutions, max pooling and skip connections. This architecture allows the U‐Net to maintain spatial information, making it effective even with smaller training datasets.^17^

In a U‐Net architecture, the left side of the U, or the encoder (contracting path), is responsible for the initial down sampling of the input image and allows the system to know what the object is within an image. The resolution of the input image is down sampled using a 2 × 2 max pooling unit which halves the resolution at every successive level of the U‐Net. As the resolution halves the number of feature channels doubles. This technique allows the U‐Net to learn more complex relationships within the image input. As we reach the bottom of the U‐Net, the right side of the U‐Net, or the decoder (expansive path), uses deconvolutions to up sample the images back to their original input resolution. At each level, the resolution doubles and the number of feature channels halves. This upsampling technique allows the U‐Net to know where the object is within an image and its size. Skip connections transfer feature maps from the encoder to the decoder at matching spatial scales. These connections allow the decoder to incorporate both high‐level and low‐level features, which improves the precision and localisation of the output.

In the article by Cao et al.,^18^ DL was used to assess accuracy of an AI model that automatically segments and quantifies body composition using CT of the lumbar spine in colorectal cancer patients. This study used a two‐dimensional U‐Net architecture. Body composition analysis has become increasingly significant, as it has been reported to be associated with clinical outcomes such as survival and has also been identified as an effective predictor of chemotherapy toxicity responses. The study aimed to evaluate the accuracy of their AI‐generated model for the automated quantification of body composition from L3 CT slices and compare its accuracy to the ground truth acquired from manual readings by experienced segmentation‐trained clinicians with consultant surgeon and radiologist oversight. They used a training dataset of 270 CT slices and validation datasets of 68 slices derived from 116 colorectal cancer patients. As with the typical development of DL models, the training dataset was used to build the U‐Net‐based DL model and the validation dataset was used to assess the performance of the final model against the ground truth (manual segmentations from expert readers). Overall, the U‐Net‐based DL model performed well in segmenting subcutaneous adipose tissue (SAT), visceral adipose tissue (VAT) and skeletal muscles. The averaged dice coefficient was 0.98 for all measurements. The differences between AI segmentation and ground truth assessments by an experienced human reader were also very small, with the average area difference being less than 5 cm^2^ and the average radiodensity difference of less than 1 Hounsfield unit (HU). The time saving is useful in a clinical setting with the study finding the AI model (0.17 ± 0.04 s) performing 4000 times faster per slice than a human reader (829.23 ± 127.09 s). However, the impact of different HU from varying kVp and CT systems was not considered in this study.

In the second article of this edition, Zhang et al.^19^ aimed to evaluate the automatic segmentation performance of MRI rectal tumours based on CNNs and propose a novel CNN model (named AttSEResUNet) with spatial attention and channel attention. This was compared to a standard U‐Net, ResUNet and U‐ with AG module. A total of 65 patients were enrolled in the study. Forty‐five patients were used for training the U‐Net models and the remaining 20 were used for testing and validating the systems. The authors used T2‐weighted (T2‐w) imaging as this is widely available and most used clinically for preoperative staging and treatment response evaluation. Two radiologists with 7 and 15 years of experience were asked to manually segment the tumour volumes on T2‐w imaging. The proposed algorithm (AttSEResUNet) performed well in comparison to observer 1 and 2 contours with a dice similarity coefficient (DSC) of 0.839 and 0.856 respectively. It also had a perfect lesion recognition rate of 100% and a false recognition rate of 0. It outperformed the other three models in all metrics when compared to the ground truth observer contours. The limitation of this study is the small sample size of only 65 patients. However, the authors performed data enhancement to increase the number of samples during the training phase of creating the model. Again, similarly to Cao et al.,^18^ the impact of image acquisition variability was not assessed in this study. MRI is more flexible compared to CT in terms of image acquisition, and different scanners, different field strengths and parameters can impact the quality of the scans acquired which could impact the final output from the U‐Net system.

## Conclusion

Deep learning is a promising and exciting method for enhancing quantitative image analysis in cancer. The literature shows the advantages of DL methods, particularly U‐Net and CNNs in semantic image segmentation. The role of image segmentation for automatic contouring has the potential to speed up and automate many workflows that have traditionally been time‐consuming and laborious. With all new technologies, caution is needed to ensure there is no generalisation of results. The impact of image quality acquired from an imaging modality using a different scanner or imaging protocol must be investigated further to ensure results from DL systems are accurate, consistent and reproducible.

## Conflict of Interest

The authors declare no conflict of interest.

## Data Availability

Data sharing is not applicable to this article as no new data were created or analyzed in this study.
